# Case Report: A case of Felty’s syndrome with initial presentation of pleural effusion and subsequent *Escherichia coli* pyothorax

**DOI:** 10.3389/fphar.2025.1529621

**Published:** 2025-04-04

**Authors:** Haifeng Yang, Weiqin Wang, Yongfeng Liu, Yibo Yang, Yiliang Su

**Affiliations:** ^1^ Department of Pulmonay and Critical Care Medicine, Tongren Hospital, Shanghai Jiao Tong University School of Medicine, Shanghai, China; ^2^ School of Clinical Medicine, Shanghai University of Medicine & Health Sciences, Shanghai, China

**Keywords:** Rheumatoid arthritis, Felty’s syndrome, pleural effusion, pyothorax, *Escherichia coli*, medical thoracoscopy

## Abstract

**Background:**

Pleural effusion is a common disease in respiratory medicine, and rheumatoid arthritis (RA) is one of the causes of pleural effusion. Felty’s syndrome is a special manifestation of RA. We report a case of Felty’s syndrome with pleural effusion as the initial symptom and the subsequent diagnosis and treatment of the patient’s secondary pyothorax.

**Case presentation:**

We report the case of a 78-year-old male who was admitted to the hospital after more than 1 year of discovered pleural effusion. After consultation with the Department of Rheumatology and Immunology, and considering the patient’s symptoms of joint deformity and swelling pain, combined with decreased white blood cells, decreased platelets, and splenomegaly, the patient was diagnosed with RA Felty’s syndrome. The patient was treated with iguratimod and methylprednisolone. Three months later, the patient was diagnosed as *Escherichia coli* empyema. The patient was treated with intravenous and sensitive anti-infection therapy combined with medical thoracoscopy, achieving good therapeutic effects.

**Conclusion:**

Felty’s syndrome is a rare manifestation of RA. Pleural effusion may be the initial manifestation of RA, and secondary infections are prone to occur during the treatment of RA and Felty’s syndrome. The combination of sensitive antibiotics and medical thoracoscopy can effectively treat secondary pyothorax.

## Introduction

Pleural effusion is a common disease in respiratory medicine. Rheumatoid arthritis (RA) is one of the causes of pleural effusion. Felty’s syndrome is a special manifestation of RA, characterized by RA symptoms along with splenomegaly, leukopenia, and thrombocytopenia. It is extremely rare for pleural effusion to be the initial presentation of Felty’s syndrome, and secondary *Escherichia coli* pyothorax is even more uncommon. Here, we report a case of Felty’s syndrome with pleural effusion as the initial symptom and the subsequent diagnosis and treatment of the patient’s secondary pyothorax.

## Case report

The patient, a 78-year-old male, admitted to the hospital due to the discovery of pleural effusion for more than a year.

**FIGURE 1:**
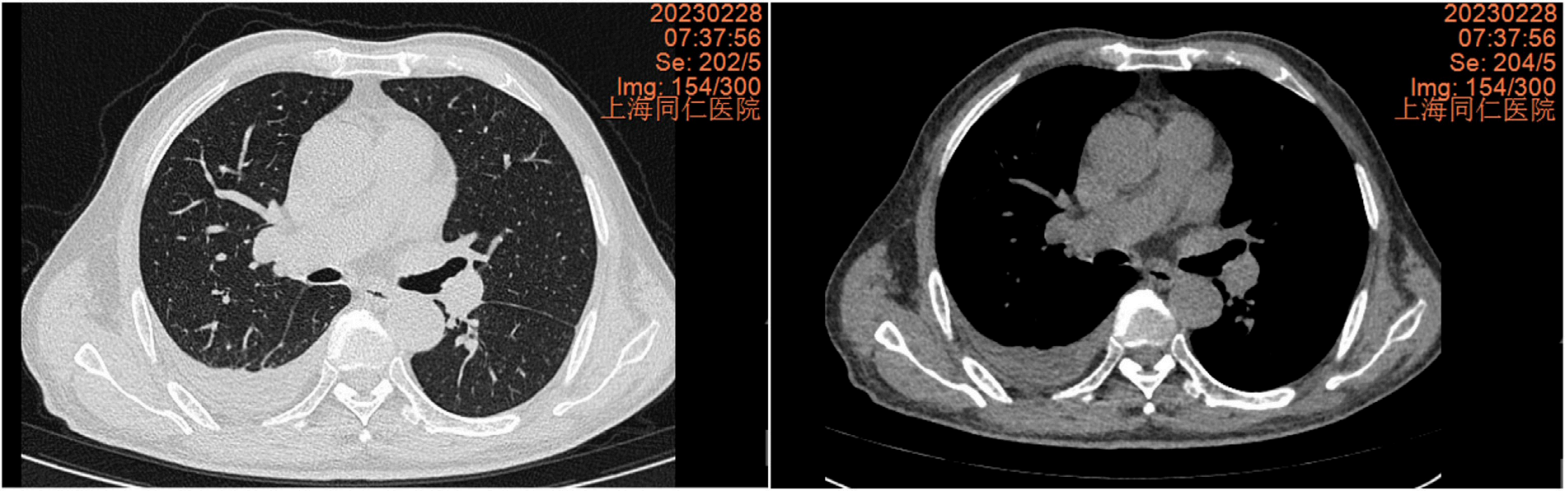
Chest CT scan from 28 February 2023, showing right pleural effusion and incomplete right lower lung atelectasis.

**FIGURE 2:**
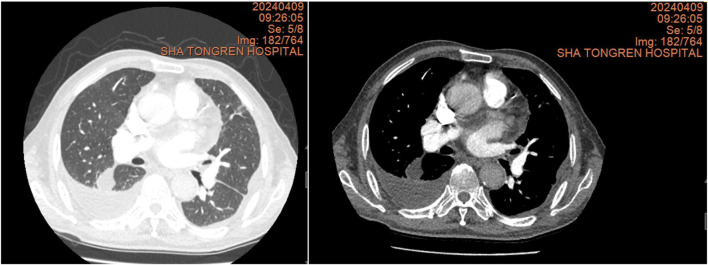
Chest CT scan from 9 April 2024, showing a significant increase in right pleural effusion compared to 28 February 2023, with a small amount of loculated effusion.

In February 2023, a chest CT scan revealed right-sided pleural effusion with incomplete expansion of the right lower lung and a small amount of inflammation in both lungs, as well as thickening of both pleural membranes (Figure 1). The patient did not exhibit symptoms such as cough, expectoration, or shortness of breath, so no further diagnosis or treatment was given. In April 2024, a chest CT scan showed right-sided pleural effusion with incomplete expansion of the right lower lung, chronic inflammation in both lungs, thickening of both pleural membranes, and splenomegaly (Figure 2). The patient was admitted to the respiratory department for further diagnosis and treatment. The patient denied a history of hypertension, diabetes, or rheumatic immune disease such as RA.

Physical examination: The patient was conscious and calm, with slightly decreased respiratory sounds in the right lower lung and no dry or wet rales. The abdomen was soft without tenderness, and the liver was not enlarged. The spleen was slightly enlarged upon palpation, and there was no edema in the lower extremities. The joints of both hands were swollen and deformed, and there was tenderness in both elbow joints.

**FIGURE 3:**
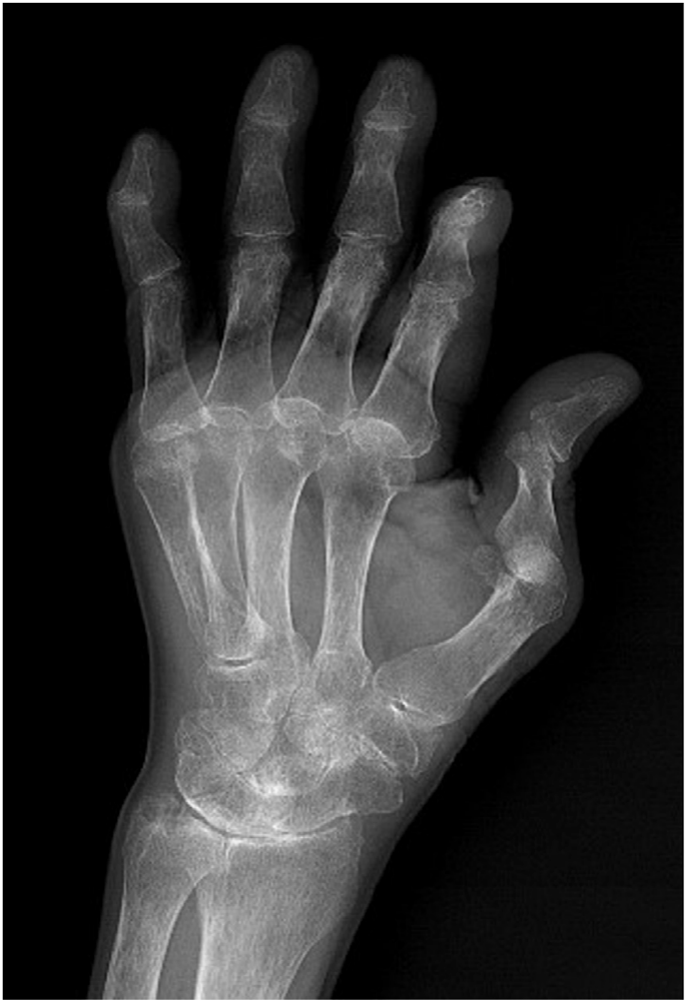
Left hand anteroposterior view from April 2024, showing swelling and deformation of the finger joints, consistent with RA bone manifestations.

Treatment Course: Further examinations were conducted after admission. Yellow turbid pleural effusion was aspirated through thoracentesis. The pleural effusion is exudative. Analysis showed a white blood cell (WBC) count of 31 × 10^6^/L, with normal lactate dehydrogenase (LDH) and glucose levels. Blood analysis revealed a WBC count of 2.78 × 10^9^/L, hemoglobin (Hb) of 88 g/L, platelets (PLT) of 68 × 10^9/L^, rheumatoid factor of 167 IU/mL, erythrocyte sedimentation rate (ESR) of 7.1 mm/h, negative antinuclear antibody (ANA), anti-cyclic citrullinated peptide (CCP) antibody of 325 U/mL, IgA of 4.57 g/L, IgG of 23.5 g/L, IgM of 2.38 g/L, IgE of 403 IU/mL (all elevated to varying degrees), and albumin of 28.7 g/L. CD4^+^ and CD8^+^ were normal. Blood QuantiFERON-TB Gold In-Tube (QFT) and Xpert MTB/RIF tests were negative. Adenosine deaminase (ADA) in pleural effusion was normal. Tumor markers in pleural effusion were normal, and no tumor cells were found. The patient exhibited RA symptoms with joint deformity and swelling. Hand X-rays showed swelling and deformity of the finger joints with bone destruction (Figure 3). A consultation with the rheumatology and immunology department, combined with leukopenia, thrombocytopenia, and splenomegaly, led to a diagnosis of Felty’s syndrome with rheumatoid arthritis.

After the diagnosis of Felty’s syndrome with rheumatoid arthritis, the patient was treated with iguratimod 25 mg twice daily orally and methylprednisolone 20 mg once daily intravenously. The patient was discharged after stabilization and continued with iguratimod 25 mg twice daily orally and methylprednisolone 20 mg once daily orally.

In July 2024, the patient developed cough, expectoration of white sticky mucus, a small amount of blood-streaked sputum, right back pain, and fever. A chest CT scan on August 7 showed bilateral lung inflammation, right lower lung encapsulated pleural effusion with partial atelectasis. Thoracentesis was performed on August 12, and pleural effusion analysis showed no tumor cells. The pleural effusion analysis revealed a WBC count of 255,264 × 10^6^/L, LDH of 34,734 IU/L, glucose <1.1 mmol/L, and *E. coli* culture, suggesting encapsulated pyothorax. The methylprednisolone was discontinued, and the patient was treated with antibiotics such as cefoperazone/sulbactam, piperacillin/tazobactam, and metronidazole. Despite treatment, the patient continued to experience cough, expectoration, back pain, fever, and blood-streaked sputum, with minimal improvement in symptoms. Rheumatoid factor was 132 IU/mL, ESR was 109.4 mm/h, ANA was negative, and anti-CCP antibody was 497 U/mL.

**FIGURE 4:**
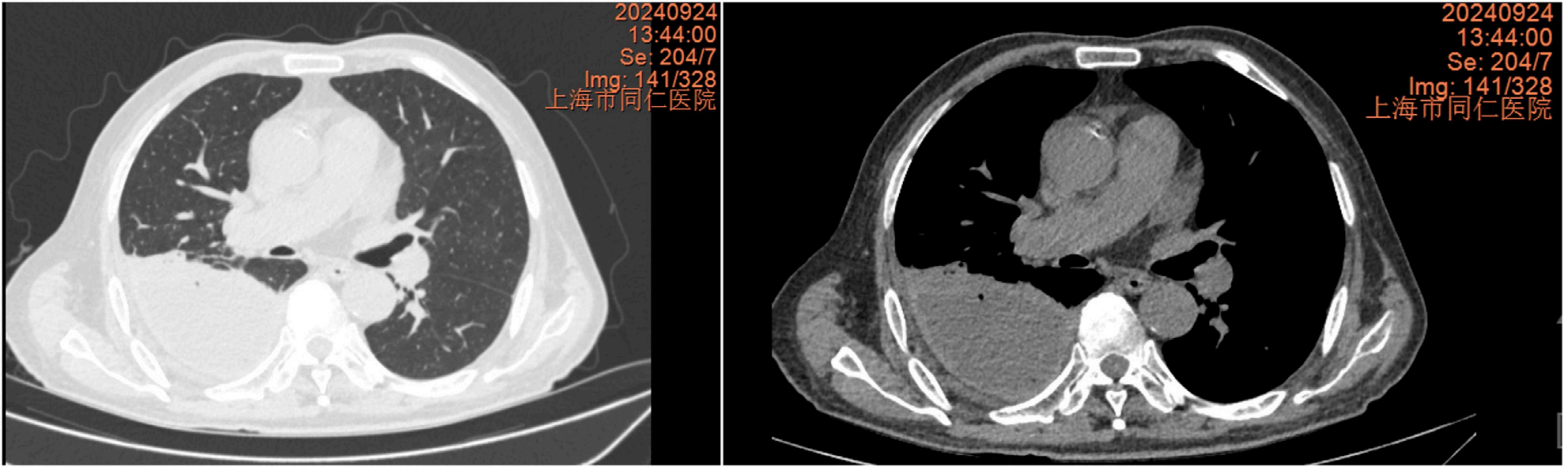
Chest CT scan from 24 September 2024, showing increased pleural effusion, pleural thickening, and bilateral lung inflammation.

**FIGURE 5:**
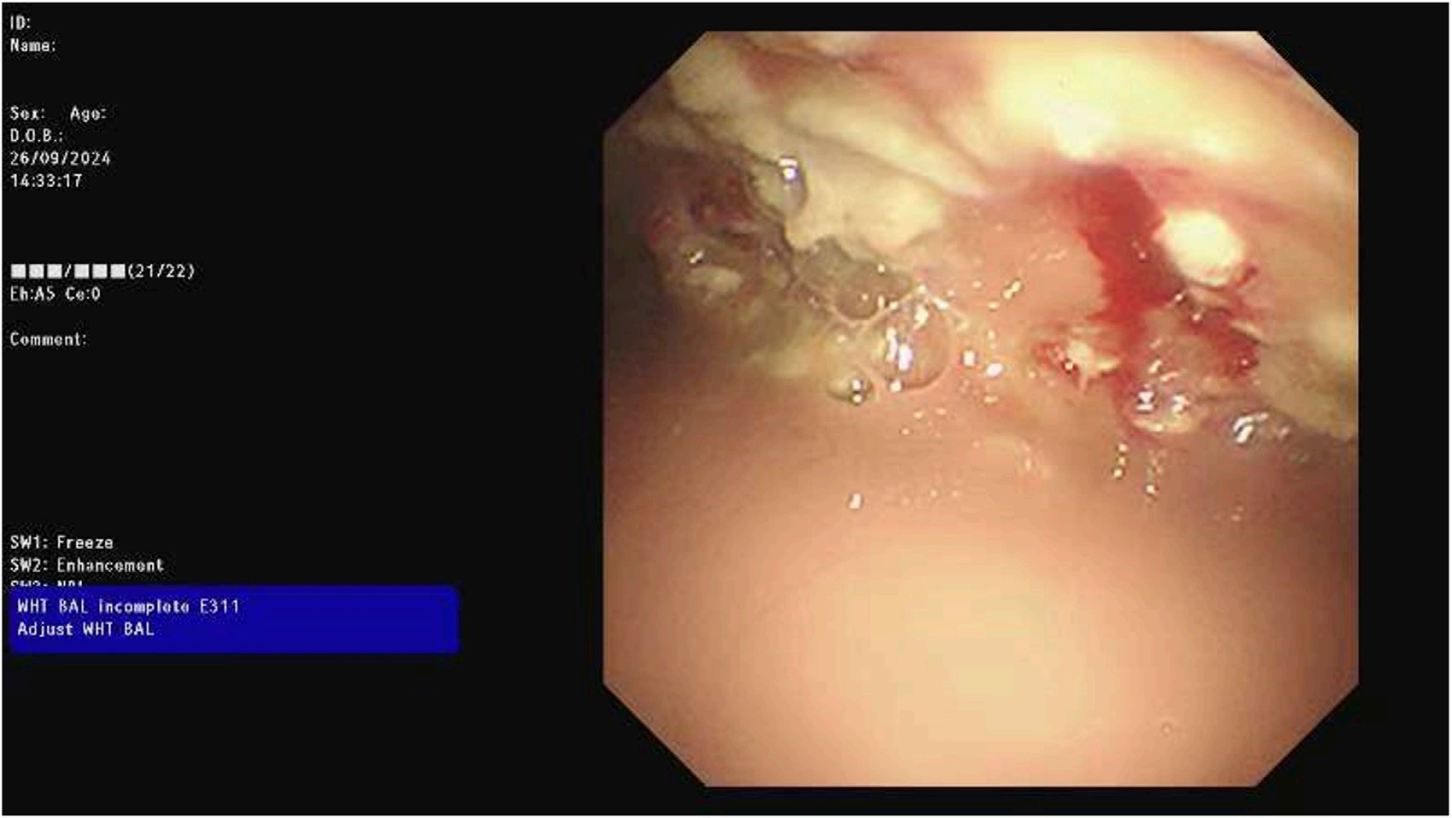
Intraoperative findings during medical thoracoscopy on 26 September 2024, showing purulent pleural effusion, widespread adhesions, septations, white necrotic pus exudates, and unclear local pleural structures.

**FIGURE 6:**
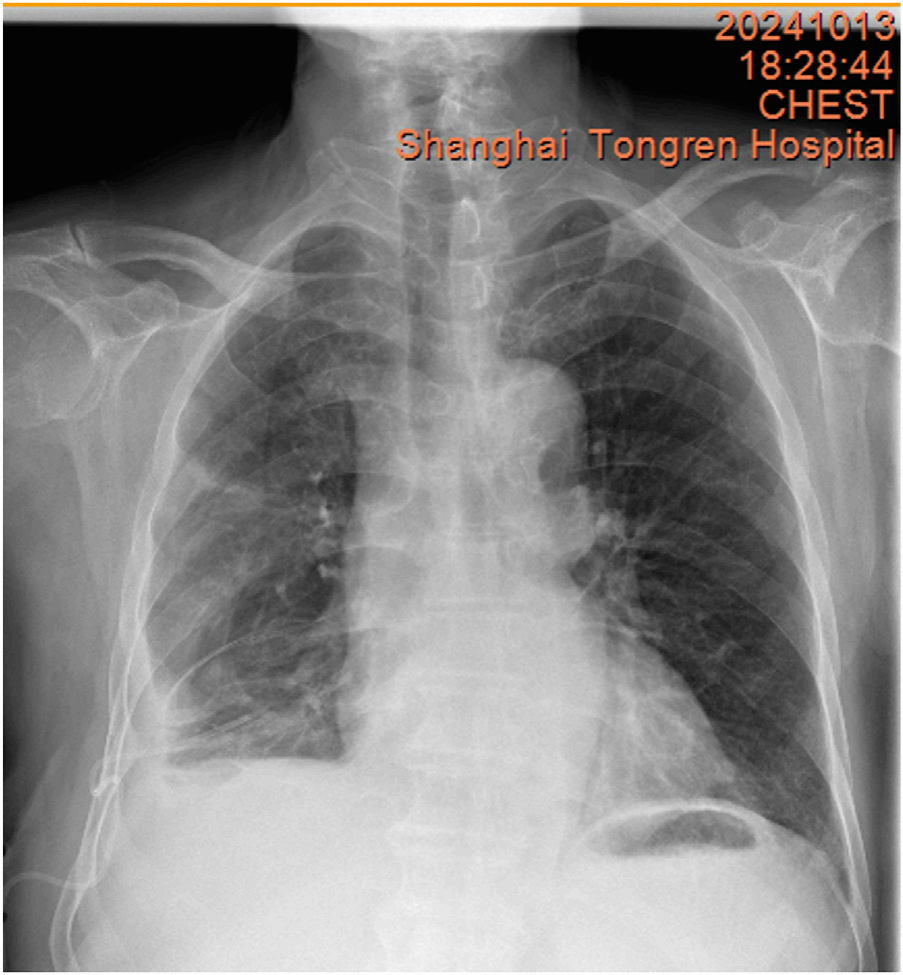
Chest X-ray on 13 October 2024, showing a decrease in right pleural effusion.

**FIGURE 7:**
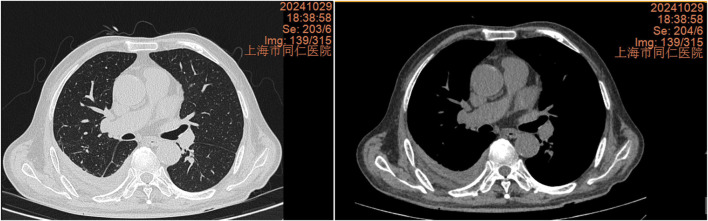
Chest CT scan 2 weeks after discharge on 29 October 2024, showing a significant reduction in right pleural effusion.

The chest CT scan on September 24 showed increased pleural effusion, pleural thickening, and bilateral lung inflammation (Figure 4). On September 26, medical thoracoscopy and pleural lysis were performed. Intraoperative findings included extensive pleural adhesions and septations, white necrotic pleural pus, and unclear local pleural structure (Figure 5). Adhesions were separated, necrotic tissue was removed, and pleural effusion was aspirated. Pathological examination showed fibrous tissue with degenerative necrosis, massive neutrophil infiltration, and local abscess formation. Pleural effusion analysis showed a WBC count of 423,420 × 10^6^/L, LDH of 6,000 IU/L, and glucose of 0.2 mmol/L. The pleural effusion culture was still positive for *E. coli*, sensitive to meropenem and amikacin. Therefore, the antibiotics were adjusted to meropenem plus amikacin. After 2 weeks of treatment, the patient’s cough and expectoration significantly improved, and fever resolved. A chest X-ray on October 13 showed a significant reduction in right-sided pleural effusion (Figure 6). The patient was discharged and continued with faropeneme 200 mg thrice daily orally. A chest CT scan on October 29 after discharge showed a significant reduction in right-sided pleural effusion (Figure 7), and the patient had no symptoms of cough, expectoration, or fever (Table 1).

**TABLE 1:** Diagnosis and treatment timeline.

Time	Treatments	Respiratory symptoms	Examinations
28 Feb 2023			Chest CT scan: right pleural effusion
9 Apr 2024			Chest CT scan: significant increase in right pleural effusion
10 Apr 2024			Pleural effusion: exudative, WBC count of 31 × 10^6^/L, with normal LDH and glucose levels
12 Apr 2024	Iguratimod 25 mg bid po Methylprednisolone 20 mg Qd ivgtt		
26 Apr 2024	Iguratimod 25 mg bid po Methylprednisolone 20 mg Qd po		
3 Jul 2024		Cough, white mucous sputum, minor hemoptysis, right back pain, and fever	
8 Aug 2024			Chest CT scan: bilateral lung inflammation, right lower lung encapsulated pleural effusion with partial atelectasis
12 Aug 2024	Thoracentesis Discontinue methylprednisolone Cefoperazone/sulbactam ivgtt 14 days Piperacillin/tazobactam plus metronidazole ivgtt 28 days		Pleural effusion analysis: WBC count of 255,264 × 10^6^/L, LDH of 34,734 IU/L, glucose <1.1 mmol/L, and *Escherichia coli* culture
24 Sep 2024		Cough, expectoration, right back pain, fever, and minor hemoptysis	Chest CT scan: increased pleural effusion, pleural thickening, and bilateral lung inflammation
26 Sep 2024	Medical thoracoscopy and pleural lysis, meropenem plus amikacin ivgtt 18 days		Pleural effusion analysis: WBC count of 423,420 × 10^6^/L, LDH of 6,000 IU/L, and glucose of 0.2 mmol/L. Positive for *Escherichia coli*, sensitive to meropenem and amikacin
13 Oct 2024	Discharge Faropenem 200 mg tid po 15 days	Cough and expectoration significantly improved, fever resolved	Chest X-ray: significant reduction in right-sided pleural effusion
29 Oct 2024		No symptoms of cough, expectoration, or fever	Chest CT scan: significant reduction in right pleural effusion

## Discussion

Felty’s syndrome is a rare special manifestation of RA, mostly occurring in elderly males and accounting for about 1% of RA patients. It typically presents in the late stage of RA, characterized by typical RA symptoms along with splenomegaly, leukopenia, thrombocytopenia, and anemia. Patients often have significant systemic symptoms, more pronounced joint inflammation and destruction, and liver and spleen enlargement, often accompanied by hypersplenism and liver cirrhosis with portal hypertension. A very small number of Felty’s syndrome patients have mild arthritis manifestations (Chavalitdhamrong et al., 2009), and pleural effusion is even rarer, with only sporadic reports of large pleural and pericardial effusions after methotrexate treatment for RA (Whitfield et al., 2020).

Pleural effusion has numerous causes, with lung infections or tumors also being potential causes. In this case, the pleural effusion lasted for a long time without initial symptoms such as cough, expectoration, dyspnea, or chest pain. Pleural effusion analysis excluded primary bacterial or tuberculous infections and tumor-related causes. RA can affect the respiratory system, leading to interstitial lung disease, subpleural rheumatoid nodules, or pleural effusion due to pleurisy. Pleural effusion is often exudative, with increased WBC count, increased protein and LDH levels, decreased glucose levels, and increased cholesterol levels in pleural effusion, which may also present as chylothorax (Nagahata and Hagino, 2024). In this case, pleural effusion is exudative, the WBC count was elevated, but glucose and LDH levels were normal, partially consistent with the characteristics of pleural effusion caused by RA. The bacteria, tuberculosis, and tumor tests in pleural effusion are also normal. So we believe that the initial pleural effusion in the patient is caused by RA. The proportion of patients with RA combined with pleural effusion is only 10%. Felty’s syndrome combined with pleural effusion is even rarer.

Felty’s syndrome was suggested by older studies showing a frequent expression of HLA-DRB1 alleles, especially HLA-DRB1*04:01 and HLA-DRB1*04:04 (HLA = human leukocyte antigen). HLA-DRB1 was expressed in 90% of cases with Felty’s syndrome (Starkebaum et al., 1997). But the patient refused to undergo allele test and family investigation. These are also limitations of this article.

Patients with Felty’s syndrome are prone to secondary infections due to leukopenia, most commonly affecting the respiratory tract and skin (Diaz et al., 2023). Compared to other types of RA, they are more susceptible to bacterial infections in pleural effusion, with *Staphylococcus*, *Streptococcus*, and other Gram-positive cocci being the most common pathogens, along with some Gram-negative bacilli. In this case, the patient initially had right pleural effusion with incomplete right lower lung atelectasis, which later developed into loculated effusion. The pleural effusion analysis showed an increased WBC count and *E. coli* culture, indicating secondary empyema in Felty’s syndrome.

Empyema occurring after glucocorticoid use usually affects immunocompromised patients, such as those with lung squamous cell carcinoma undergoing immunotherapy (Zhou et al., 2022), HIV patients (Kalinoski et al., 2020), or those with COVID-19 pneumonia (Placik et al., 2020). Empyema is closely related to decreased immune function due to the patient’s underlying disease. Although the patient had normal CD4^+^ and CD8^+^ levels after diagnosis with Felty’s syndrome. He was prescribed methylprednisolone, the possibility of immune dysfunction caused by oral glucocorticoids leading to secondary empyema cannot be ruled out. Therefore, glucocorticoids were discontinued immediately after diagnosing empyema, and the patient was treated with intravenous and sensitive anti-infection therapy combined with medical thoracoscopy, achieving good therapeutic effects.

The treatment principles for pleural effusion combined with Felty’s syndrome are basically the same as those for pleural effusion combined with RA. In cases of concurrent secondary infection or empyema, it is necessary to identify the specific bacterial infection through pleural fluid culture and administer corresponding sensitive antibiotics in sufficient doses for an adequate duration. Active drainage of pus and systemic supportive treatment are also crucial. In this case, the pleural fluid culture revealed *E. coli*, and based on the drug sensitivity results, we administered Meropenem and Amikacin, achieving positive outcomes. Sullivan (Sullivan et al., 2022) reported a middle-aged man with RA, without pulmonary involvement, on disease-modifying antirheumatic drugs presented with right sided pleuritic chest pain due to recurrent, right sided, loculated pleural effusion. Non-typhi *Salmonella* was isolated from pleural fluid sampling and the patient was successfully treated with open thoracotomy with decortication and 6 weeks of antibiotic therapy. Tang et al. (2021) reported a 47-year-old man with progressive RA on immunosuppressive therapy who was found to have a left-sided pleural effusion. He was treated with levofloxacin and methylprednisolone. He presented a month later and was found to have a large left-sided thick-walled fluid collection found to be an empyema. A chest tube was placed, and fluid culture grew *Fusobacterium* nucleatum. The patient was successfully treated with intrapleural fibrinolytic therapy and amoxicillin-clavulanic acid. These cases (include ours) are all opportunistic infections. Treatments are all based on anti-infection therapy. Some cases involve the use of surgery, while others involve the use of intrapleural fibrinolytic therapy. We also involve the use of medical thoracoscopy.

Felty’s syndrome, as a rare manifestation of RA, can exhibit clinical features of respiratory system involvement in RA. Although the patient in this case denied any history of rheumatic immune system diseases and tested negative for ANA, the patient’s swollen and deformed hands, tender elbows, significantly elevated rheumatoid factor, and positive anti-CCP antibodies strongly suggested a diagnosis of RA. The pleural effusion was considered a pulmonary manifestation of RA. Secondary infections are prone to occur during the treatment of RA and Felty’s syndrome. Close monitoring for infectious manifestations in patients is therefore imperative. For patients developing secondary pyothorax, the administration of sensitive antibiotics is crucial. When combined with medical thoracoscopic intervention, superior therapeutic outcomes can be achieved.

## Data Availability

The original contributions presented in the study are included in the article/supplementary material, further inquiries can be directed to the corresponding authors.
